# Supplementary material for: Genome-Wide Identification and Characterization of WRKY Transcription Factors in Betula platyphylla Suk. and Their Responses to Abiotic Stresses
Source: Int J Mol Sci. 2023 Oct 8;24(19):15000. doi: 10.3390/ijms241915000 (PMC10573109; doi:10.3390/ijms241915000)
Supplement: Supplementary file 1 [file ijms-24-15000-s001.zip › Figure S1a Multiple sequence alignment analysis of Classó±.pdf]

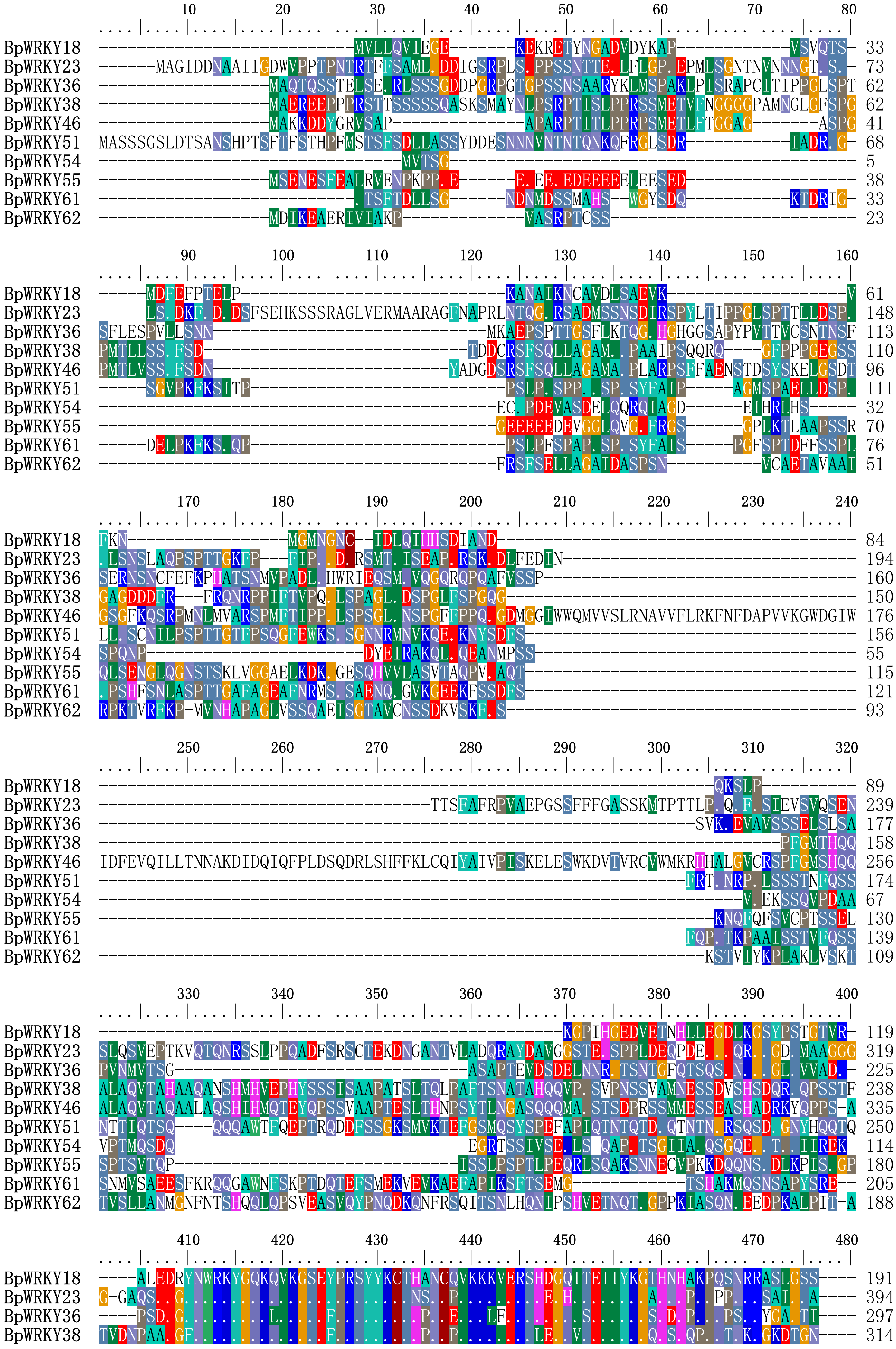
BpWRKY18
BpWRKY23
BpWRKY36
BpWRKY38
BpWRKY46
BpWRKY51
BpWRKY54
BpWRKY55
BpWRKY61
BpWRKY62

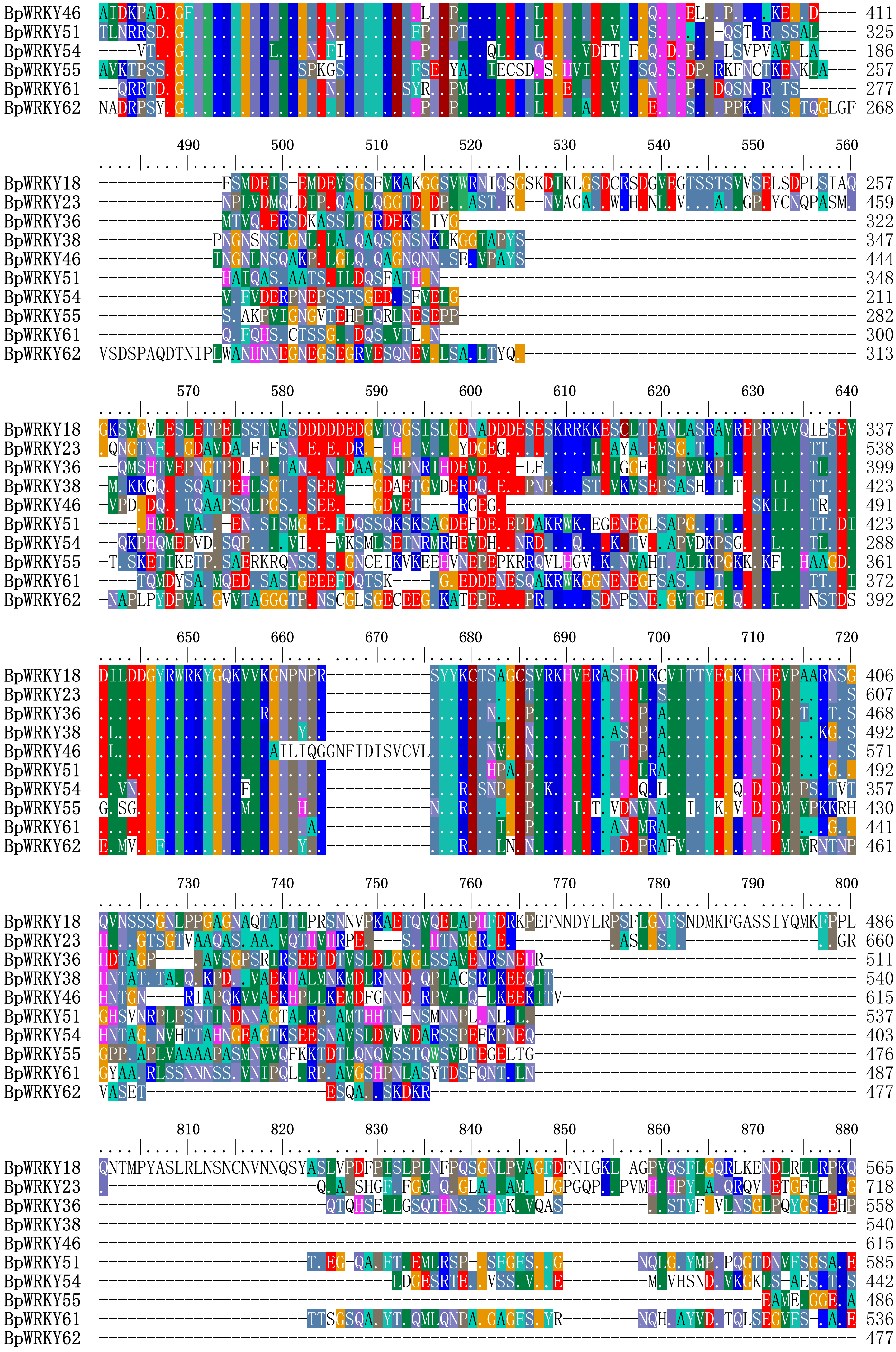

```
                   890       900       910
          ....|....|....|....|....|....|....|...
BpWRKY18  EQKDDSLYDACLPIVDHGNVTSSLYHQVMGRFPP----  599
BpWRKY23  .P.AEPMSEPG.NLSNG----T.V.QHI.S.L.LGPQM  752
BpWRKY36  NGSYPFPQNMGKILTGP---------------------  575
BpWRKY38  --------------------------------------  540
BpWRKY46  --------------------------------------  615
BpWRKY51  .PR..MFLESL.A-------------------------  598
BpWRKY54  .GS.TVCL.MVGCS------------------------  456
BpWRKY55  MESART.LSIGFE.KPC---------------------  503
BpWRKY61  .P--NTFF.SF.SEDY----------------------  550
BpWRKY62  --------------------------------------  477
```
