# Supplementary material for: Genome-Wide Identification and Characterization of WRKY Transcription Factors in Betula platyphylla Suk. and Their Responses to Abiotic Stresses
Source: Int J Mol Sci. 2023 Oct 8;24(19):15000. doi: 10.3390/ijms241915000 (PMC10573109; doi:10.3390/ijms241915000)
Supplement: Supplementary file 1 [file ijms-24-15000-s001.zip › Figure S1b Multiple sequence alignment analysis of Class ó≥.pdf]

```
                   10        20        30        40        50        60        70        80
           ....|....|....|....|....|....|....|....|....|....|....|....|....|....|....|....|
BpWRKY1    --------------------------------------------------------------------------------  1
BpWRKY2    --------------------------------------------------------------------------------  1
BpWRKY5    --------------------------------------------------------------------------------  1
BpWRKY8    --------------------------------------------------------------------------------  1
BpWRKY10   --------------------------------------------------------------------------------  1
BpWRKY11   --------------------------------------------------------------------------------  1
BpWRKY12   --------------------------------------------------------------------------------  1
BpWRKY13   ---------------------------------------MAEG-RLSMGKETVKDSYVKP-----------NHTTWKQNI  29
BpWRKY14   MSVYSLLQVLPRAPLLNGDVILPAEKTAGPLFSFLTKISFSKDPPPPPYKPYKESLQATKNICYQYSNTTTTTLSLRLSL  80
BpWRKY15   --------------------------------------------------------------------------------  1
BpWRKY16   --------------------------------------------------------------------------------  1
BpWRKY19   --------------------------------------------------------------------------------  1
BpWRKY20   --------------------------------------------------------------------------------  1
BpWRKY21   --------------------------------------------------------------------------------  1
BpWRKY22   --------------------------------------------------------------------------------  1
BpWRKY25   --------------------------------------------------------------------------------  1
BpWRKY26   --------------------------------------------------------------------------------  1
BpWRKY27   --------------------------------------------------------------------------------  1
BpWRKY28   --------------------------------------------------------------------------------  1
BpWRKY29   --------------------------------------------------------------------------------  1
BpWRKY30   -----------------------------------------------------------------------------MCS  3
BpWRKY31   --------------------------------------------------------------------------------  1
BpWRKY32   --------------------------------------------------------------------------------  1
BpWRKY33   ---------------------------------------MDKGWGLTLDSAESPGSFLLN-------------KNKRSHD  28
BpWRKY35   --------------------------------------------------------------------------------  1
BpWRKY37   --------------------------------------------------------------------------------  1
BpWRKY39   --------------------------------------------------------------------------------  1
BpWRKY40   --------------------------------------------------------------------------------  1
BpWRKY42   --------------------------------------------------------------------------------  1
BpWRKY43   --------------------------------------------------------------------------------  1
BpWRKY44   --------------------------------------------------------------------------------  1
BpWRKY45   -----------------------------------------------------------------------------MDG  3
BpWRKY48   --------------------------------------------------------------------------------  1
BpWRKY49   --------------------------------------------------------------------------------  1
BpWRKY50   --------------------------------------------------------------------------------  1
BpWRKY52   ---------------------------------------MAKGSGLSIDSPDPFGFFPHRPIVLNSFPEDNNNNNRNIHK  41
BpWRKY53   ---------------------------------------MAKGSGLSIDSPDPFGFFPHRPIVLNSFPEDNNNNNRNIHK  41
BpWRKY59   --------------------------------------------------------------------------------  1
BpWRKY60   --------------------------------------------------------------------------------  1
BpWRKY64   --------------------------------------------------------------------------------  1

                   90       100       110       120       130       140       150       160
           ....|....|....|....|....|....|....|....|....|....|....|....|....|....|....|....|
BpWRKY1    -----------------MDSNYCLDTSLGLNLNSFPQHMN----------------------------------------  23
BpWRKY2    -----------------.RN.F.FLILP--FIVL.NSCNS----------------------------------------  21
BpWRKY5    -----------------------MSDEPKDHVYYHDLFQDIQK-------------------------------------  20
BpWRKY8    ----------------------------MEGWDLQAVVR-----------------------------------------  11
BpWRKY10   --------------------------------------------------------------------------------  1
BpWRKY11   -----------------.EAAKE.GSGH.DG----VLREE----------------------------------------  19
BpWRKY12   ---------------------------MESSWPENSSSK-----------------------------------------  12
BpWRKY13   MDLS--------------N..PF.NCGDHEH.YLGSSNGK------PIVKEMDFFAADK---------------------  68
BpWRKY14   SLSLLVMENQHRREFTFLR.RDF.GRHNNPGGPDRTSENSMEQSQPPMIKEMDFFSGTS-------------------TT  141
BpWRKY15   ---------------------MEKKEAIKTEEYQTVGSSSFS--------------------------------------  21
BpWRKY16   --------------------------------------------------------------------------------  1
BpWRKY19   --------------------------------------------------------------------------------  1
BpWRKY20   --------------------------------------------------------------------------------  1
BpWRKY21   --------------------------------------------------------------------------------  1
BpWRKY22   ----------------------------M..QGL.DDQEVPSQ-------------------------------------  15
BpWRKY25   --------------------------------------------------------------------------------  1
BpWRKY26   --------------------------------------------------------------------------------  1
BpWRKY27   ---------------------------MEEDWDLQAVVRGCAS-------------------------------------  16
BpWRKY28   ---------------------------MASTWSDVSEDD-----------------------------------------  12
BpWRKY29   -----------------------MEEVMASTWSDVSEDD-----------------------------------------  16
BpWRKY30   LFLQRMENYQGDLTDIVRA.GGAYACGG.VAGD.E.ISDWQFHTDPINFSPVVEKPRDNFG-------------------  64
BpWRKY31   --------------------------------------------------------------------------------  1
BpWRKY32   --------------------------------------------------------------------------------  1
BpWRKY33   MIF------------PGIQFPVT.GR-RDEHAAPP.SDD.-----RVTVGEVDFFADKGRSAGHDDHDDNIHGSKTLRVS  90
BpWRKY35   ----------------------MMSSHSDRHEP.TSVEN-------SVIKEFDFFS------------------------  27
BpWRKY37   -----------------------M.DGDKSDPGNEFTSDDSS--------------------------------------  19
```

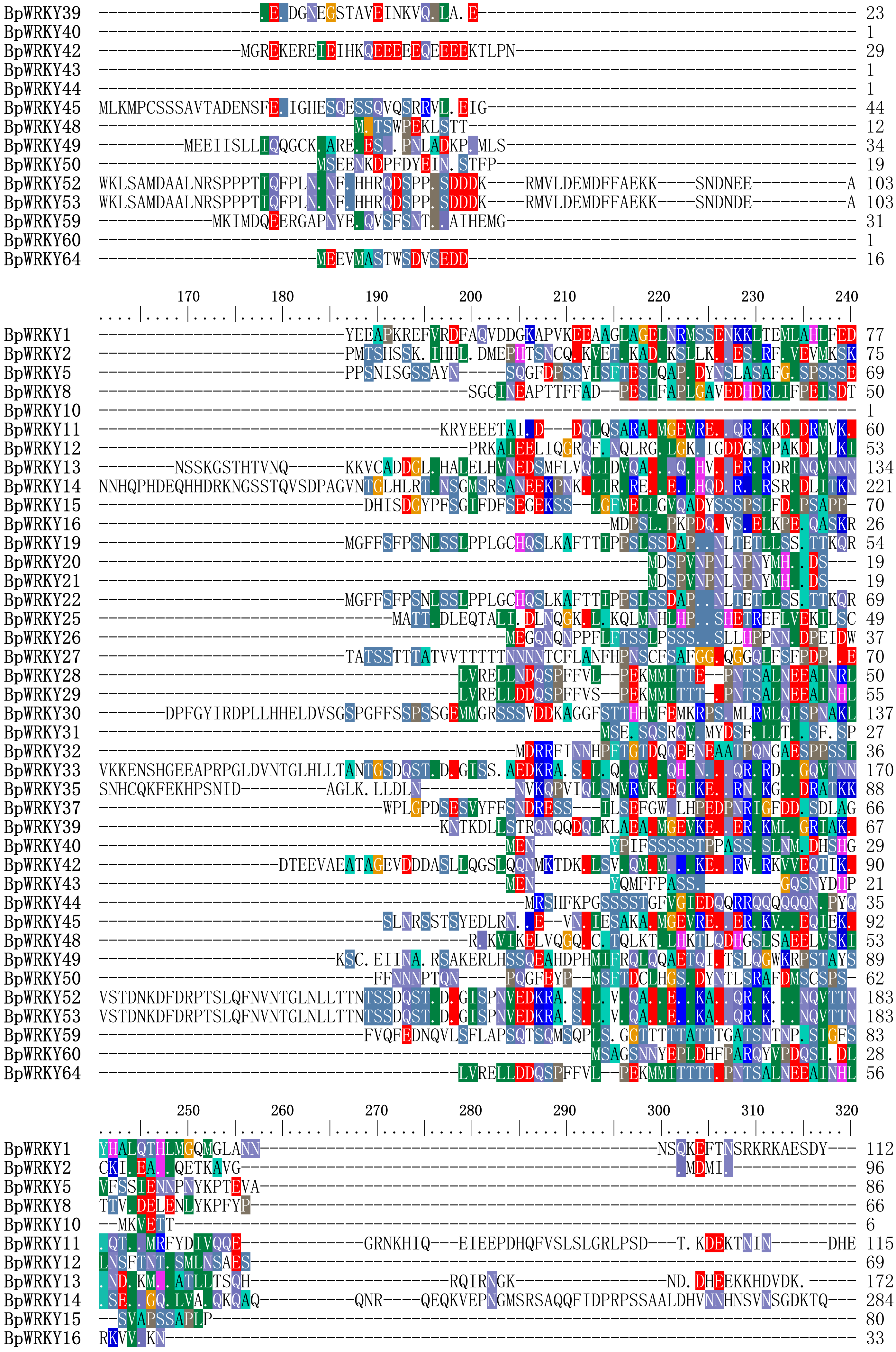

BpWRKY39 23
BpWRKY40 1
BpWRKY42 29
BpWRKY43 1
BpWRKY44 1
BpWRKY45 44
BpWRKY48 12
BpWRKY49 34
BpWRKY50 19
BpWRKY52 103
BpWRKY53 103
BpWRKY59 31
BpWRKY60 1
BpWRKY64 16
170 180 190 200 210 220 230 240
BpWRKY1 77
BpWRKY2 75
BpWRKY5 69
BpWRKY8 50
BpWRKY10 1
BpWRKY11 60
BpWRKY12 53
BpWRKY13 134
BpWRKY14 221
BpWRKY15 70
BpWRKY16 26
BpWRKY19 54
BpWRKY20 19
BpWRKY21 19
BpWRKY22 69
BpWRKY25 49
BpWRKY26 37
BpWRKY27 70
BpWRKY28 50
BpWRKY29 55
BpWRKY30 137
BpWRKY31 27
BpWRKY32 36
BpWRKY33 170
BpWRKY35 88
BpWRKY37 66
BpWRKY39 67
BpWRKY40 29
BpWRKY42 90
BpWRKY43 21
BpWRKY44 35
BpWRKY45 92
BpWRKY48 53
BpWRKY49 89
BpWRKY50 62
BpWRKY52 183
BpWRKY53 183
BpWRKY59 83
BpWRKY60 28
BpWRKY64 56
250 260 270 280 290 300 310 320
BpWRKY1 112
BpWRKY2 96
BpWRKY5 86
BpWRKY8 66
BpWRKY10 6
BpWRKY11 115
BpWRKY12 69
BpWRKY13 172
BpWRKY14 284
BpWRKY15 80
BpWRKY16 33

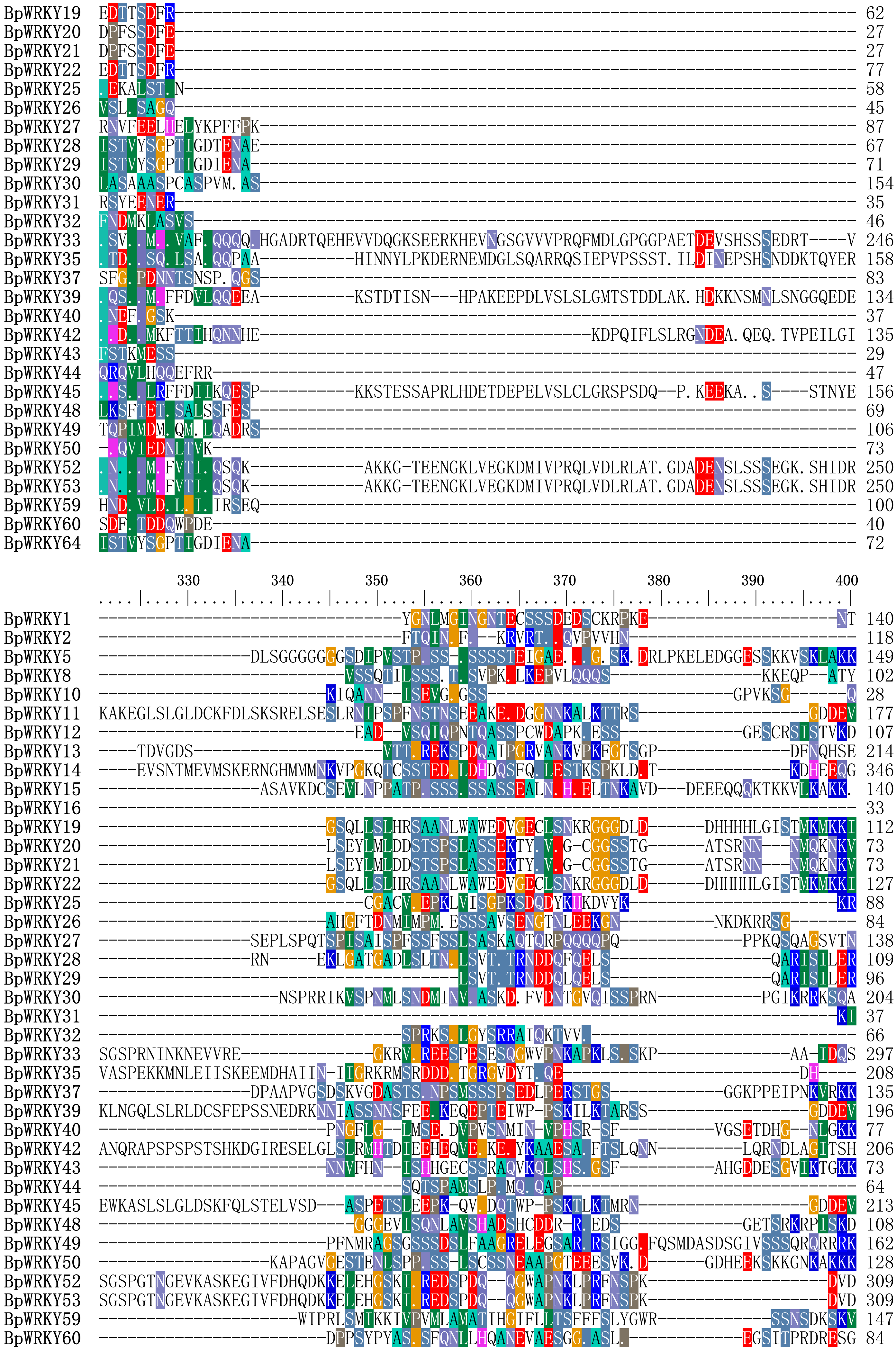

BpWRKY19 EDTTSDFR-- 62
BpWRKY20 DPFSSDFE-- 27
BpWRKY21 DPFSSDFE-- 27
BpWRKY22 EDTTSDFR-- 77
BpWRKY25 .EKALST.N--- 58
BpWRKY26 VSL.SAGQ-- 45
BpWRKY27 RNVFEELHELYKPFFPK--- 87
BpWRKY28 ISTVYSGPTIGDTENAE--- 67
BpWRKY29 ISTVYSGPTIGDIENA-- 71
BpWRKY30 LASAAASPCASPVM.AS--- 154
BpWRKY31 RSYEENER-- 35
BpWRKY32 FNDMKLASVS-- 46
BpWRKY33 .SV..M..VAF.QQQQ.HGADRTQEHEVVDQGKSEERKHEVNGSGVVVPRQFMDLGPGGPAETDEVSHSSSEDRT----V 246
BpWRKY35 .TD..SQ.LSA.QQPAA----------HINNYLPKDERNEMDGLSQARRQSIEPVPSSST.ILDINEPSHSNDDKTQYER 158
BpWRKY37 SFG.PDNNTSNSP.QGS--- 83
BpWRKY39 .QS..M.FFDVLQQEEA----------KSTDTISN---HPAKEEPDLVSLSLGMTSTDDLAK.HDKKNSMNLSNGGQEDE 134
BpWRKY40 .NEF.GSK-- 37
BpWRKY42 ..D..MKFTTIHQNNHE-------------------------------KDPQIFLSLRGNDEA.QEQ.TVPEILGI 135
BpWRKY43 FSTKMESS-- 29
BpWRKY44 QRQVLHQQEFRR-- 47
BpWRKY45 ..S..LRFFDIIKQESP----------KKSTESSAPRLHDETDEPELVSLCLGRSPSDQ--P.KEEKA..S----STNYE 156
BpWRKY48 LKSFTET.SALSSFES-- 69
BpWRKY49 TQPIMDM.QM.LQADRS--- 106
BpWRKY50 -.QVIEDNLTVK-- 73
BpWRKY52 .N...M.FVTI.QSQK-----------AKKG-TEENGKLVEGKDMIVPRQLVDLRLAT.GDADENSLSSSEGK.SHIDR 250
BpWRKY53 .N...M.FVTI.QSQK-----------AKKG-TEENGKLVEGKDMIVPRQLVDLRLAT.GDADENSLSSSEGK.SHIDR 250
BpWRKY59 HND.VLD.L.I.IRSEQ--- 100
BpWRKY60 SDF.TDDQWPDE-- 40
BpWRKY64 ISTVYSGPTIGDIENA-- 72
330 340 350 360 370 380 390 400
BpWRKY1 -------------------------------YGNLMGINGNTECSSSDEDSCKRPKE-------------------NT 140
BpWRKY2 -------------------------------FTQIN.F.--KRVRT..QVPVVHN----------------------- 118
BpWRKY5 ----------------DLSGGGGGGGSDIPVSTP.SS-.SSSSTEIGAE..G.SK.DRLPKELEDGGESSKKVSKLAKK 149
BpWRKY8 ---------------------------VSSQTILSSS.T.SVPK.LKEPVLQQQS----------------KKEQP--ATY 102
BpWRKY10 -------------------------KIQANN--ISEVG.GSS----------------------GPVKSG-------Q 28
BpWRKY11 KAKEGLSLGLDCKFDLSKSRELSESLRNIPSPFNSTNSEEAKE.DGGNNKALKTTRS-------------------GDDEV 177
BpWRKY12 -------------------------EAD--VSQIQPNTQASSPCWDAPK.ESS--------------GESCRSISTVKD 107
BpWRKY13 ----TDVGDS-------------------VTT.REKSPDQAIPGRVANKVPKFGTSGP-----------------DFNQHSE 214
BpWRKY14 ----EVSNTMEVMSKERNGHMMMNKVPGKQTCSSTED.LDHDQSFQ.LESTKSPKLD.T--------------KDHEEQG 346
BpWRKY15 -----------------ASAVKDCSEVLNPPATP.SSS.SSASSEALN.H.ELTNKAVD---DEEEQQQKTKKVLKAKK. 140
BpWRKY16 -- 33
BpWRKY19 -------------------------GSQLLSLHRSAANLWAWEDVGECLSNKRGGGDLD------DHHHHLGISTMKMKKI 112
BpWRKY20 -------------------------LSEYLMLDDSTSPSLASSEKTY.V.G-CGGSSTG------ATSRNN---NMQKNKV 73
BpWRKY21 -------------------------LSEYLMLDDSTSPSLASSEKTY.V.G-CGGSSTG------ATSRNN---NMQKNKV 73
BpWRKY22 -------------------------GSQLLSLHRSAANLWAWEDVGECLSNKRGGGDLD------DHHHHLGISTMKMKKI 127
BpWRKY25 ------------------------------CGACV.EPKLVISGPKSDQDYKHKDVYK-------------------KR 88
BpWRKY26 -------------------------AHGFTDNMIMPM.ESSSAVSENGTNLEEKGN----------NKDKRRSG------- 84
BpWRKY27 ----------------SEPLSPQTSPISAISPFSSFSSLSASKAQTQRPQQQQPQ--------------PPKQSQAGSVTN 138
BpWRKY28 ----------------RN------EKLGATGADLSLTN.LSVT.TRNDDQFQELS--------------QARISILER 109
BpWRKY29 -----------------------------------LSVT.TRNDDQLQELS--------------QARISILER 96
BpWRKY30 -------------------NSPRRIKVSPNMLSNDMINV.ASKD.FVDNTGVQISSPRN-------------PGIKRRKSQA 204
BpWRKY31 --KI 37
BpWRKY32 -------------------------------SPRKS.LGYSRRAIQKTVV.-------------------------------- 66
BpWRKY33 SGSPRNINKNEVVRE-------------GKRV.REESPESESQGWVPNKAPKLS.SKP----------------AA-IDQS 297
BpWRKY35 VASPEKKMNLEIISKEEMDHAIIN-IIGRKRMSRDDD.TGRGVDYT.QE-----------------------DH---- 208
BpWRKY37 ----------------DPAAPVGSDSKVGDASTS.NPSMSSSPSEDLPERSTGS-----------GGKPPEIPNKVRKK 135
BpWRKY39 KLNGQLSLRLDCSFEPSSNEDRKNNIASSNNSFEE.KEQEPTEIWP-PSKILKTARSS------------------GDDEV 196
BpWRKY40 -------------------------PNGFLG--LMSE.DVPVSNMIN-VPHSR-SF------------VGSETDHG--NLGKK 77
BpWRKY42 ANQRAPSPSPSTSHKDGIRESELGLSLRMHTDIEEHEQVE.KE.YKAAESA.FTSLQNN----------LQRNDLAGITSH 206
BpWRKY43 -------------------------NNVFHN--ISHHGECSSRAQVKQLSHS.GSF-----------AHGDDESGVIKTGKK 73
BpWRKY44 -------------------------------SQTSPAMSLP.MQ.QAP------------------------------- 64
BpWRKY45 EWKASLSLGLDSKFQLSTELVSD---ASPETSLEEPK-QV.DQTWP-PSKTLKTMRN------------------GDDEV 213
BpWRKY48 --------------------------GGEVISQNLAVSHADSHCDDR-R.EDS--------------GETSRKRPISKD 108
BpWRKY49 -------------------------PFNMRAGSGSSSDSLFAAGRELEGSAR.RSIGG.FQSMDASDSGIVSSSQRQRRRK 162
BpWRKY50 ------------------KAPAGVGESTENLSPP.SS-LSCSSNEAAPGTEEESVK.D------GDHEEKSKKGNKAKKK 128
BpWRKY52 SGSPGTNGEVKASKEGIVFDHQDKKELEHGSKI.REDSPDQ--QGWAPNKLPRFNSPK---------------------DVD 309
BpWRKY53 SGSPGTNGEVKASKEGIVFDHQDKKELEHGSKI.REDSPDQ--QGWAPNKLPRFNSPK---------------------DVD 309
BpWRKY59 ---------------------WIPRLSMIKKIVPVMLAMATIHGIFLLTSFFFSLYGWR------------SSNSDKSKV 147
BpWRKY60 -------------------------DPPSYPYAS.SFQNLLHQANEVAESGG.ASL.------------EGSITPRDRESG 84

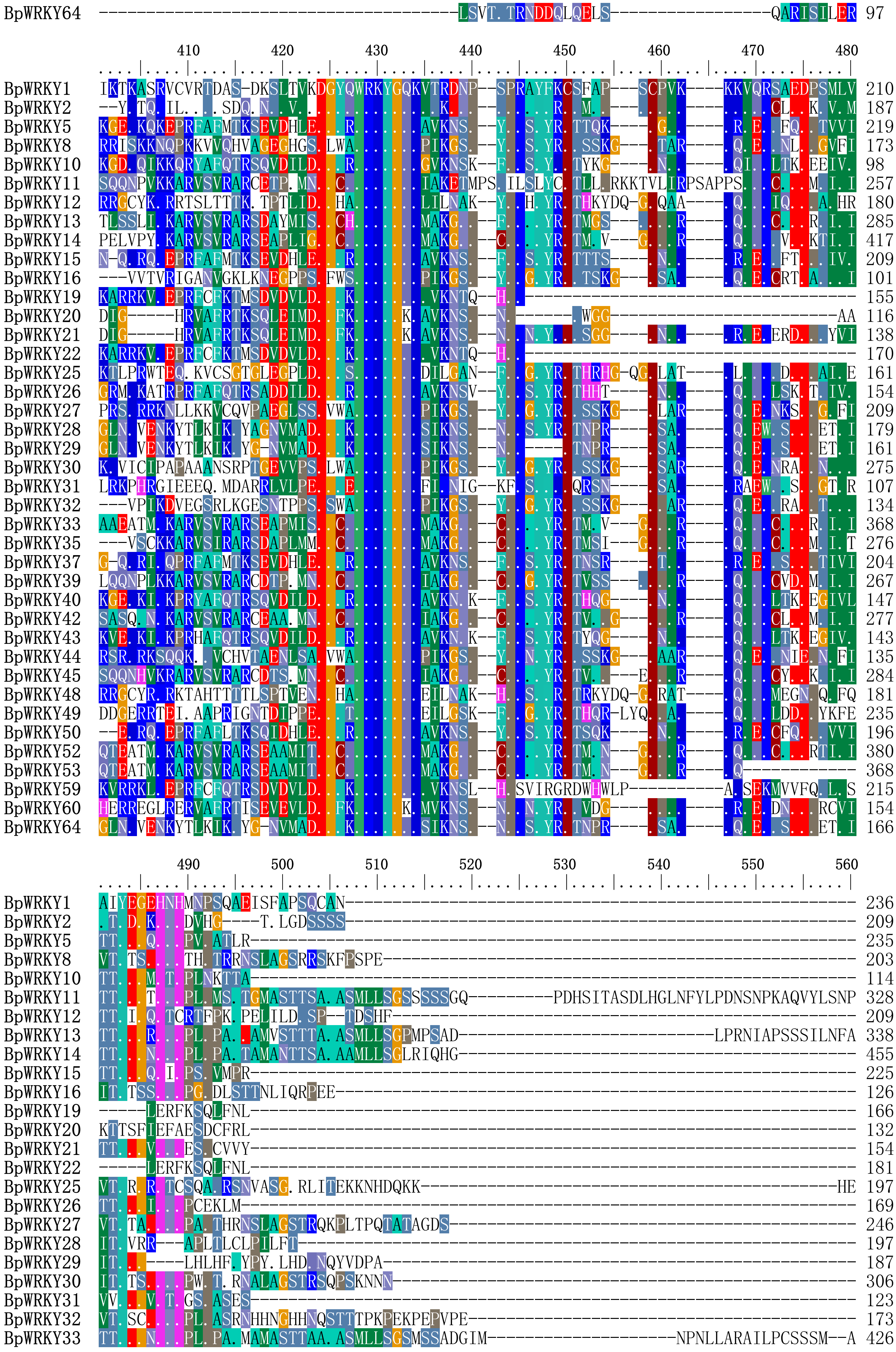
BpWRKY64
BpWRKY1
BpWRKY2
BpWRKY5
BpWRKY8
BpWRKY10
BpWRKY11
BpWRKY12
BpWRKY13
BpWRKY14
BpWRKY15
BpWRKY16
BpWRKY19
BpWRKY20
BpWRKY21
BpWRKY22
BpWRKY25
BpWRKY26
BpWRKY27
BpWRKY28
BpWRKY29
BpWRKY30
BpWRKY31
BpWRKY32
BpWRKY33
BpWRKY35
BpWRKY37
BpWRKY39
BpWRKY40
BpWRKY42
BpWRKY43
BpWRKY44
BpWRKY45
BpWRKY48
BpWRKY49
BpWRKY50
BpWRKY52
BpWRKY53
BpWRKY59
BpWRKY60
BpWRKY64

```
BpWRKY35  ST...N...PL.PP.TFMASTTSA.VSMLLSGSTTSN-------------------------------------------  313
BpWRKY37  TT...Q.C.HTVGFPR----------------------------------------------------------------  220
BpWRKY39  TT...T...PL.IS.TAMASTTSA.ASMLQSRSSSSSQLGGLGTSATAPLSTSTANNLHGFNFSTVSQNSRPPQFYIPNS  347
BpWRKY40  TT...M.S.PIEKST-----------------------------------------------------------------  162
BpWRKY42  TT...T...PL.VG.TAMASTASA.SFMLLDSSNPTSSFTQAS---------------------------------LPYH  324
BpWRKY43  TT...V.T.PIEKPT-----------------------------------------------------------------  158
BpWRKY44  VT.T...T.PR.THRNSLAGSTRNKCSAPHKSIDKENSASLS-----------------------------TANIGSCSS  186
BpWRKY45  TT...T...PL.VS.AAMASTTSA.ASMLLSGSSMSQPGLG-----LTPTATNAAPTLNGLNFNLLD-NSKVKQFYLPNS  358
BpWRKY48  TT.I.H.TCREIIKTPQIITE.HAWELTNFF-------------------------------------------------  212
BpWRKY49  VT.R.D.TCHMS.T.PSIPP.-----------------------------------------------------------  256
BpWRKY50  TT...Q...HY.ATLR----------------------------------------------------------------  212
BpWRKY52  TT...N.S.PL.PN.MAMASTTSS.AKMLLSGSMSSADGLM--------------------NSNFLTRTLLPCSSSM--A  438
BpWRKY53  ----------------------NTPAMLLKHG-------------------------------NNLSLS-----------  384
BpWRKY59  CVNL..KDMSLWLP.KN---------------------------------------------------------------  232
BpWRKY60  TT...V...QSSF-------------------------------------------------------------------  167
BpWRKY64  IT..----LHLHF.YPY.LHD.NQYVDPA---------------------------------------------------  192

                   570       580       590       600       610       620       630       640
          ....|....|....|....|....|....|....|....|....|....|....|....|....|....|....|....|
BpWRKY1   ----LGSVHLSSSMRSSAPRVTLDLIQP-----------------------------ALCGAKN--SIQEDDESPRA---  278
BpWRKY2   ----SDT.ARG.MANIIS.PRMANNNN.-----------------------------F.QPMALDL.LSGPHQEN.R---  253
BpWRKY5   -GNAA.MFPP.MFTPPTGGGP.SFP----------------------------------HHHQDLF---FRWR-------  270
BpWRKY8   -KKGNF.-PTTPL.AAAIEDEFVQSHAS---------GKNHEEEEMVQGNYDQGNNVISMPDNIILDNEIFFP.LED---  269
BpWRKY10  -DSFEQIFRQMQTYAPLYMK------------------------------------------------------------  133
BpWRKY11  SYSSSS.FPTITLDLT.TSSSSSSSH.FNKFSSYYPPRYNN----------YSPTSLNFSNSSD-HQPNAISWGYAN---  394
BpWRKY12  --VLSFDNTFTNKHNHPVHPFLSSSF.S---------VKQEHKEEMPSD--DVTHNQSSSSDYLVSPD.TAFD.------  270
BpWRKY13  TLSTSAPFPTVTLDLTDH.----HNSS.NNLP--------------------QVFGQS.PNQS.YFGLYGSQGMDLP---  391
BpWRKY14  NPIGFCTISHHHPRLDTE.QPMQPTTPFHRIPAVPSSA--------------TIFPL..H.FQHLLG-HPVYSF.SS---  517
BpWRKY15  -QILA.APPD.GFSGGR.ASGFAMP..RSICHYQQQQRQPISHIHALSPSNFANYNGLTNNPAGNY---LREIRFCN---  298
BpWRKY16  -PEQTH.TEDLPTPTKEEQQEIAEEEEEKQNDPIMSTTEKDHDFHYIQSPIRGCLDIIVNQGEEDPFTVHLEKTHETMG-  204
BpWRKY19  -L------------------------------------------------------------------------------  167
BpWRKY20  -G-----------LK.CT--------------------------------------------------------------  138
BpWRKY21  -YNQTPLMVP.GWTLQASSV------------------------------------------------------------  173
BpWRKY22  -L------------------------------------------------------------------------------  182
BpWRKY25  EKLPQEI.RNFAARLTVKTEEY.EAREDDIFPSFSIGSENEEANIFCESMLENNFMAS.SPTFLSPATS.SNMF.MSPCQ  277
BpWRKY26  -ETLTPLLKQMQFLSRF---------------------------------------------------------------  185
BpWRKY27  -KKLNSTKPS.PATSMEEDL.VPQSTTT---------DSKEEREDLVE---DEEEDEFGLSDVAMSDD--FFVGLEG---  308
BpWRKY28  ---RPK--PVCG.SNQE.QEEYVLSQR.--------NPMKPNKLKKP--TKAQQILRL.RY-------------------  243
BpWRKY29  -IKKHKNSTI.RAQAHE.QQTQDAQES.-----------AYLTTGPVLSP--PEDQGGIIM.SQGLLEDVVPMMIRNP---  251
BpWRKY30  -NGAAANSKNKPTSPKEEQKDQSSNEANVSPSTTTSTACVKEEIDHDHQDIEKMDDAD.ISTGTDHHGFPYRPAMP.G--  383
BpWRKY31  ---ASSAHSGT.ANQYDLLTQVFGDRS-------------------------------TYNDHHDN--------------  156
BpWRKY32  -KQLDQETTS.AVLADLGDESLMLSDEFGWFSDMETTSSTVLESPIFADSSVGGDADLAMFFPMREEDESLYADLGELPE  252
BpWRKY33  TISASAPFPTVTLDLTHS.NPLQFQRT.AQFPVHFAGQPQSFGSVPTQPLPGQGFGQ..YNQSKFAGL.LSQDMGPSQLA  506
BpWRKY35  NIFNPP.NSGLFPLM.KY.STVAT.STAPHPTITLDLT---------------HTPQRNMQSLQQRRALDPPPLFMSS---  376
BpWRKY37  -G---.VISHETFAGQFH.P.SQFYYP--------TGAPLPQENPFIITQSQQIPGEAGDESSRAMPGPTP---------  279
BpWRKY39  SFSTSN.HPTVTLDLTT..STSSSFERFSSTPRYPSTRLN-----------FSSSSLDQPNTLQNHR.NGGYPGYSNYGY  416
BpWRKY40  -DNFEHILTQMQIFS.Y---------------------------------------------------------------  178
BpWRKY42  MINPSSHPSNILTTINPNDPSKGIVLDLTTNPYDHHHHPPQFPMPAGPSSYSSSSSSS.H.FA.WMHGKQSYHNNATN--  402
BpWRKY43  -DNFEHILSQMQIYAPF---------------------------------------------------------------  174
BpWRKY44  PQSATSLSPTTPLTVQTDEEAIIANKKSNEEENKIMIDGNDKESEEMADDYDEDDDEI.IPNMAMTQDIFLGLQELG---  263
BpWRKY45  S-SPAPLCPTVTLDLTTS.FSSST--LFNRLPSSFASNPI-----------FPSKSLSFSSLESNILPTTWGAGYPSYSA  424
BpWRKY48  --VNSE.TTIP.KQDGAVS----SSAAT---------IKQESKEEALS---DLTDNLPSL.-IHLWPDLN.F.LSGP---  270
BpWRKY49  --AAATDMTTQPRSS..I.LSSWLSMEYLG------------PRGGGSGSSSGIV.S.SGGGG.GDGAGP.TTRTG-  318
BpWRKY50  -GNAA.MLSP.LLASA.GG-P.TFPQA------LFSQLLPTNNNQAVDPNYMFYGNLMTQHPQQQQ---QQLQ-------  274
BpWRKY52  TISASAPFPTVTLDLTQS.NPLQFQRPSTQFQIPFANP-------SAASLLPQIFGQ..QNQSKFSGL.MSQDMDQG---  508
BpWRKY53  ------PISHCYIGFNTI.QPITIPKAINPIPNPICQS-------LRRLIAASNIWPSPSKPIKFSGL.MSQDMDQG---  448
BpWRKY59  --KIFHILWKPFVGSNLRSYYRCTHNN------------------------CRVKKRVERLSEDCRMVITTY.GRHN---  283
BpWRKY60  --------------------------------------------------------------------------------  167
BpWRKY64  -IKKPKKSTI.QAQAHE.QQTQDAQES.-----------AYLTTGPVLSP--LEDREGIIM.SQGLLEDVVPMMIRNP---  256

                   650       660       670       680       690       700       710       720
          ....|....|....|....|....|....|....|....|....|....|....|....|....|....|....|....|
BpWRKY1   ----------------------------PLQHFLVQ------QMASSLTRDPNFTAALAAAISGRILDSTTLN-------  317
BpWRKY2   ----------------------------.F.N.VEDNHRNIAEY.A...K..S........VASS.TQQPKVSKI-----  300
BpWRKY5   ---------------------------------.K---------------------------------------------  272
BpWRKY8   -----------------------------FAEP--AVDGWFLDQLLDNCPL.W.IDKST.VGDDHC---------------  304
BpWRKY10  --------------------------------------------------------------------------------  133
BpWRKY11  ------------QVIPYDR---NQQNMGRQSN------YAIAAATKAI.A..S.QS.....LTTI.GTGN----------  443
BpWRKY12  -------------------------------FYNMTVLSSPVESDDNGVLVGSVDHFADDDLM.AFSF------------  307
BpWRKY13  ----------------------LLATNRI.SPPSLLDADAVSSATAAI.AN..........ASI.DN-------------  437
BpWRKY14  ------------------------NKQAVD.VGQ..QPSMVHTLSAAIAA.........V.TI.GAPARS.DGNNGAT  573
```

```
BpWRKY15  -----------------------------TGSA.LRDHGLLQDIVP.HMLKEE--------------------------- 322
BpWRKY16  ----------------------------L.LDEEPLCYSQLMNFSTPFSEEND.FDE.EELPTSSFT.FMRSNFSDERI 256
BpWRKY19  -------------------------------------------------------------------------------- 167
BpWRKY20  -------------------------------------------------------------------------------- 138
BpWRKY21  -------------------------------------------------------------------------------- 173
BpWRKY22  -------------------------------------------------------------------------------- 182
BpWRKY25  MN---------------------SYGLGYNVQTSESDLTDIVSAPT.V.NS.ILNLDFSLDKVDHLDPNFPFDNPDFFY- 335
BpWRKY26  -------------------------------------------------------------------------------- 185
BpWRKY27  -----------------------------FAGP--AAGDCFPDHFPATFAL..WV.NN.G-------------------- 337
BpWRKY28  -------------------------------------------------------------------------------- 243
BpWRKY29  -----------------------------PNNTFSNSSSCSSYRSPPTSPSSLSWCTSYS.TCFD.GI------------ 290
BpWRKY30  ----------------------------TSNCAQSSDQDFFADLGEIEADPLDLLFTQ.FSGDDDHDHQEKDSKALMDPF 435
BpWRKY31  -------------------------------------------------------------------------------- 156
BpWRKY32  CSVVFRWRG--------------TRSRDL.TRVFGPHYPPLPRLSTVKVVPFLSFV..CETANMVVVEQLVKSLTPLIFF 318
BpWRKY33  HHASSHP---------------PLQQPQQQ.QPSLA--DTVSAATAAI.A.............SI.-SGAHP.----NNT 564
BpWRKY35  ------------------------MLPTLFPAEKDRHRSMVDMVSAAIAT.H.........A.SIMRA.QSNSHGGHDTS 432
BpWRKY37  --------------------------------QFPSNEGLLGDIVPPNM.NR---------------------------- 299
BpWRKY39  NSNTSTTGNQPFQEHLYQANYMNNQNNGFHHDQQPALEENIVAATKAIASN.K.QSV....LTSYVG..RENQGVGDQIS 496
BpWRKY40  -------------------------------------------------------------------------------- 178
BpWRKY42  ------------------------NLFPSPRRVDEENNKSLAENVTAMAS..K.RV.V....TSLMNKESHTGPHVGTTT 458
BpWRKY43  -------------------------------------------------------------------------------- 174
BpWRKY44  -----------------------------YSSPGGGSGDNFSDGGRANSGSSWPPCGSS..AIATTGAAMNGC------- 307
BpWRKY45  VPFNKTN---ASSMNLFQSYLEKTHQASSQ.S-------LTESLTKAIAT....QS.I..T..SIVGGGA.HDSQGGGEP 494
BpWRKY48  -----------------------------IGSDNGDVVSTMYSCTDTASLSLELDIDFDGDF.EFL-------------- 307
BpWRKY49  ------------------------------KEVEFPVVDLADA.FN.GSSSS.ISMDFLFHPTEDKWETGDKQN------ 362
BpWRKY50  ---------------------------------.PHDYGLLQDLVPPFSNKRQP-------------------------- 295
BpWRKY52  ------------------------QGQQLG.QNSLS--DTVSAATAAIAA............TSI.GSGAHT.NICGNNA 562
BpWRKY53  ------------------------QGQQLG.QNSLS--DTVSAATAAIAA............TSI.GSGAHT.NICGNNA 502
BpWRKY59  ----------------------------------------HSPCDD.NSSEHECFTSF---------------------- 301
BpWRKY60  -------------------------------------------------------------------------------- 167
BpWRKY64  -----------------------------PNNTFSN--SCSSYRSPPTSPSSLSWCTSYSTTCFD.GI------------ 293

                  730       740       750       760       770       780       790       800
          ....|....|....|....|....|....|....|....|....|....|....|....|....|....|....|....|
BpWRKY1   -------------------------------------------------------------------------------- 317
BpWRKY2   -------------------------------------------------------------------------------- 300
BpWRKY5   -------------------------------------------------------------------------------- 272
BpWRKY8   -------------------------------------------------------------------------------- 304
BpWRKY10  -------------------------------------------------------------------------------- 133
BpWRKY11  ---------------------------GCASSFLNKTH-STNSPAPG--SLMFLPPS-LPFSAPKSASTSPG-DNRDNIT 491
BpWRKY12  -------------------------------------------------------------------------------- 307
BpWRKY13  ----------------------------VRSNHSSNSHTTTT-------------------------------------- 451
BpWRKY14  SNPGL-----------------------PGSPQLPQSCTTFSTN------------------------------------ 594
BpWRKY15  -------------------------------------------------------------------------------- 322
BpWRKY16  PVVPS--------------------------------------------------------------------------- 261
BpWRKY19  -------------------------------------------------------------------------------- 167
BpWRKY20  -------------------------------------------------------------------------------- 138
BpWRKY21  -------------------------------------------------------------------------------- 173
BpWRKY22  -------------------------------------------------------------------------------- 182
BpWRKY25  -------------------------------------------------------------------------------- 335
BpWRKY26  -------------------------------------------------------------------------------- 185
BpWRKY27  -------------------------------------------------------------------------------- 337
BpWRKY28  -------------------------------------------------------------------------------- 243
BpWRKY29  -------------------------------------------------------------------------------- 290
BpWRKY30  SLFDWSGDKN---------------------------------------------------------------------- 445
BpWRKY31  -------------------------------------------------------------------------------- 156
BpWRKY32  LFLVSLLLGMNWQSVAIVVGIFEQRGVPYNVYAFVNRLHLDKEIITLGL------------------------------- 367
BpWRKY33  N---------------------------TSNNTTGNKISNFQGN------------------------------------ 581
BpWRKY35  SSK-M-----------------------PGSPQLPQSCTTFSTI------------------------------------ 452
BpWRKY37  -------------------------------------------------------------------------------- 299
BpWRKY39  SSLMKLKWGDHSRTVNHIYPSSVQNVIGCGSSYLNKSSPSINSPQQGGGGLALFPPNPLQLSASKGASTAPA-DNRDHFQ 575
BpWRKY40  -------------------------------------------------------------------------------- 178
BpWRKY42  FGPRD----------------------GETGSSSTTNNWVLESLPGNGKPIRHSP------------------------- 491
BpWRKY43  -------------------------------------------------------------------------------- 174
BpWRKY44  -------------------------------------------------------------------------------- 307
BpWRKY45  IQAVS-------------SDALTQKGKACASSFFNRFP-SSNSHTAG---LMFLQPP-LPLSISKSSSTSTSSAIRDQMN 556
BpWRKY48  -------------------------------------------------------------------------------- 307
BpWRKY49  -------------------------------------------------------------------------------- 362
BpWRKY50  -------------------------------------------------------------------------------- 295
BpWRKY52  NPTTT-----------------------TTTNSNGNSNSSFQGK------------------------------------ 583
BpWRKY53  NPTTT-----------------------TTTNSNGNSNSSFQGK------------------------------------ 523
```

```
BpWRKY59  -------------------------------------------------------------------------------- 301
BpWRKY60  -------------------------------------------------------------------------------- 167
BpWRKY64  -------------------------------------------------------------------------------- 293


          ...
BpWRKY1   --- 317
BpWRKY2   --- 300
BpWRKY5   --- 272
BpWRKY8   --- 304
BpWRKY10  --- 133
BpWRKY11  KEF 494
BpWRKY12  --- 307
BpWRKY13  --- 451
BpWRKY14  --- 594
BpWRKY15  --- 322
BpWRKY16  --- 261
BpWRKY19  --- 167
BpWRKY20  --- 138
BpWRKY21  --- 173
BpWRKY22  --- 182
BpWRKY25  --- 335
BpWRKY26  --- 185
BpWRKY27  --- 337
BpWRKY28  --- 243
BpWRKY29  --- 290
BpWRKY30  --- 445
BpWRKY31  --- 156
BpWRKY32  --- 367
BpWRKY33  --- 581
BpWRKY35  --- 452
BpWRKY37  --- 299
BpWRKY39  L-- 576
BpWRKY40  --- 178
BpWRKY42  --- 491
BpWRKY43  --- 174
BpWRKY44  --- 307
BpWRKY45  --- 556
BpWRKY48  --- 307
BpWRKY49  --- 362
BpWRKY50  --- 295
BpWRKY52  --- 583
BpWRKY53  --- 523
BpWRKY59  --- 301
BpWRKY60  --- 167
BpWRKY64  --- 293
```
