# Supplementary material for: Genome-Wide Identification and Characterization of WRKY Transcription Factors in Betula platyphylla Suk. and Their Responses to Abiotic Stresses
Source: Int J Mol Sci. 2023 Oct 8;24(19):15000. doi: 10.3390/ijms241915000 (PMC10573109; doi:10.3390/ijms241915000)
Supplement: Supplementary file 1 [file ijms-24-15000-s001.zip › Figure S1d Multiple sequence alignment analysis of Class ó⌠.pdf]

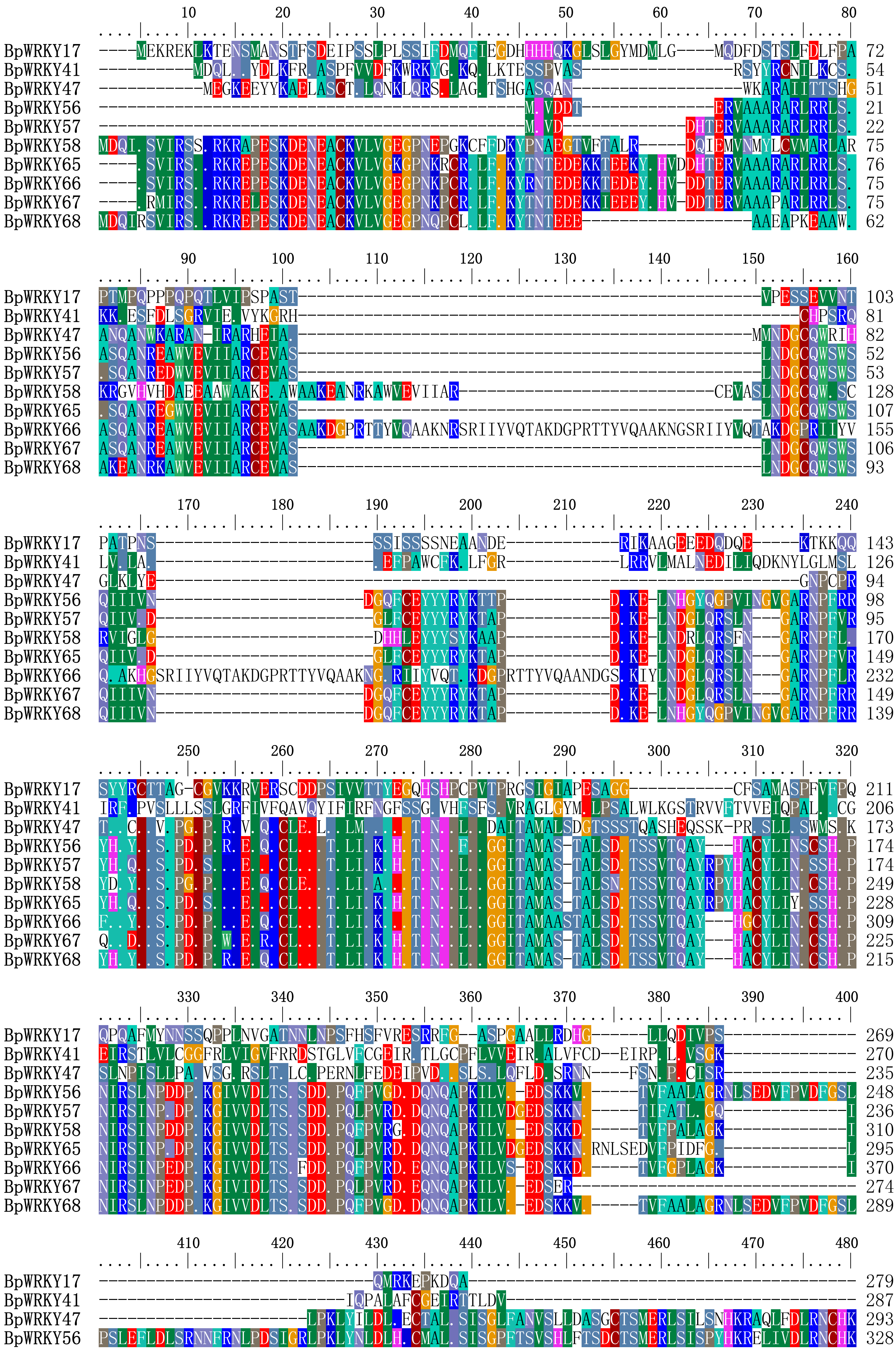

BpWRKY17
BpWRKY41
BpWRKY47
BpWRKY56
BpWRKY57
BpWRKY58
BpWRKY65
BpWRKY66
BpWRKY67
BpWRKY68

```
BpWRKY57  ET-------------------------HTNT------------------------------------------------- 242
BpWRKY58  EMVRCMHM-------------------HMNLCVVAGLRVKS--------------------------------------- 332
BpWRKY65  PSLEFLNLSRNNFRNLPDSIGRLPKLYNLDLH.CLAL.SISGPFASISHLLTSDCTSMERLSISPYHKKGLVVDLRNCHK 375
BpWRKY66  EMVRCMHM-------------------HMNLCVVAGLRVKS--------------------------------------- 392
BpWRKY67  -----------------------------RYCIR.TRR------------------------------------------ 283
BpWRKY68  PSLEFLDLSRNNFRNLPDSIGRLPKLYKLDLH.CMAL.SISG-------------------------------------- 331

                  490       500       510       520       530       540       550       560
          ....|....|....|....|....|....|....|....|....|....|....|....|....|....|....|....|
BpWRKY17  -------------------------------------------------------------------------------- 279
BpWRKY41  -------------------------------------------------------------------------------- 287
BpWRKY47  LTEIQGLENWEFASIHSEGRKILAYDFRSHFQCPSTMHDDQYRIICLPGSEVPNWFSHQRVGSSISFRVPSISNGQILRL 373
BpWRKY56  LTEIQGLENWEFACIHNPDITSHLQCPS----TMG------DGVIYLLGREVPNCFSNQTIGSSISFRVPSISKGQFIRV 398
BpWRKY57  -------------------------------------------------------------------------------- 242
BpWRKY58  -------------------------------------------------------------------------------- 332
BpWRKY65  LTEIQGLENWEFAFFHNLAYDIRSHLQY----CPSTMGDDEFGVICLAGNEVPNCFSNQTIGSSISFRVPSISKGQFIRV 451
BpWRKY66  -------------------------------------------------------------------------------- 392
BpWRKY67  -------------------------------------------------------------------------------- 283
BpWRKY68  -----------------------PFASY----CPSTMGDDKSGVICLRGSEVPNCFSNQTIGSSTSFRVPSISKGQFIRV 384

                  570       580       590       600       610       620       630       640
          ....|....|....|....|....|....|....|....|....|....|....|....|....|....|....|....|
BpWRKY17  -------------------------------------------------------------------------------- 279
BpWRKY41  -------------------------------------------------------------------------------- 287
BpWRKY47  LVCAVYATNKGSNPS-----------DVFPPCVKIQNKTRGNQHILWPTLFPAPVTCGNHFFLFPKKEDHLFLFQTPLIK 442
BpWRKY56  LVCAIYAHNARYAHYPVDEEELDWVEADLPPSVRILNKTRGNFQFFWTTYFSSPIFEGKHSFSSPKKEDHFVRYLTPLIR 478
BpWRKY57  -------------------------------------------------------------------------------- 242
BpWRKY58  -------------------------------------------------------------------------------- 332
BpWRKY65  LVCAIYAHNACYPRYPLDEEELDWVEADLPPSVRILNKTRGNFHVFSTTYFSGPIFEGKHSFPCPKK-DHFFLYLTPLIR 530
BpWRKY66  -------------------------------------------------------------------------------- 392
BpWRKY67  -------------------------------------------------------------------------------- 283
BpWRKY68  LVCVIYAHNARYARYPVDEEELDWVEADLPPSVRILNKTRGNFHVFWTKYFSGPIFEGKHSFPCPKK-DHFFLYLTPLIR 463

                  650       660       670       680       690       700
          ....|....|....|....|....|....|....|....|....|....|....|....|...
BpWRKY17  --------------------------------------------------------------- 279
BpWRKY41  --------------------------------------------------------------- 287
BpWRKY47  N-----------ELVIESGEEIEVFIELWAAAKVKKCGIHLLVDEPNVIEEDVTLNNFGGVIF 494
BpWRKY56  RKFELEGGRIFNELVMDSGDEIEVYIDFWYGSEVKKCGVHVVVDEPCSNLHDVEVM------- 534
BpWRKY57  --------------------------------------------------------------- 242
BpWRKY58  --------------------------------------------------------------- 332
BpWRKY65  NKYELEGGRIFNELVMDSADEINVHIDFWYGSEVKKCGVHVVVDEPCSNLYDAEVM------- 586
BpWRKY66  --------------------------------------------------------------- 392
BpWRKY67  --------------------------------------------------------------- 283
BpWRKY68  NKYELEGGRIFNELVMDSGDEIEVYIDFWYGSEVKKCGVHVVVDEPCSNLHDAEVM------- 519
```
