# Supplementary material for: Genome-Wide Identification and Characterization of WRKY Transcription Factors in Betula platyphylla Suk. and Their Responses to Abiotic Stresses
Source: Int J Mol Sci. 2023 Oct 8;24(19):15000. doi: 10.3390/ijms241915000 (PMC10573109; doi:10.3390/ijms241915000)
Supplement: Supplementary file 1 [file ijms-24-15000-s001.zip › Figure S2 Conserved domains of BpWRKYs protein..pdf]

A.

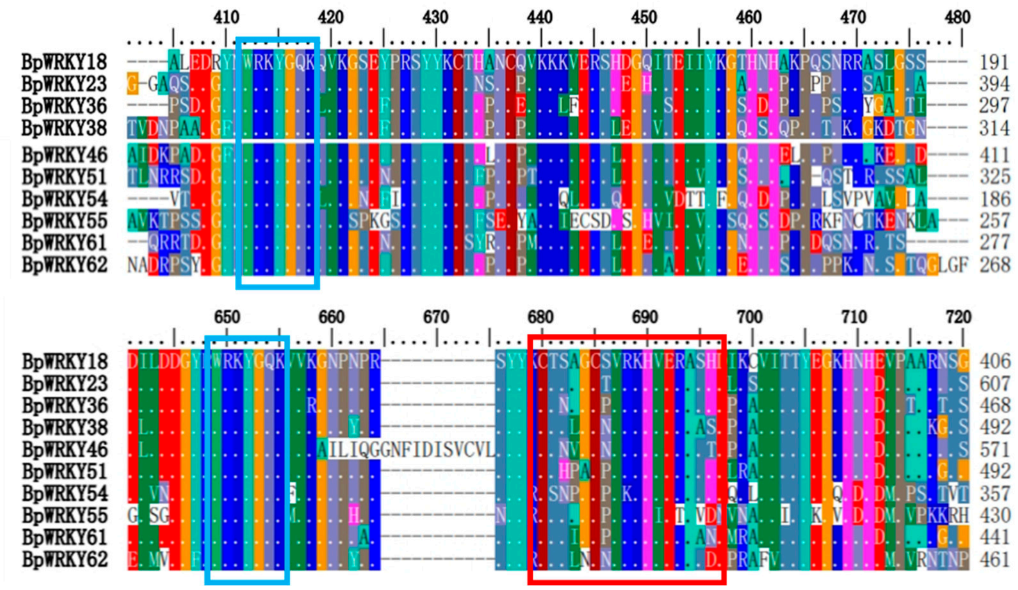

410
420
430
440
450
460
470
480
BpWRKY18 191
BpWRKY23 394
BpWRKY36 297
BpWRKY38 314
BpWRKY46 411
BpWRKY51 325
BpWRKY54 186
BpWRKY55 257
BpWRKY61 277
BpWRKY62 268
650
660
670
680
690
700
710
720
BpWRKY18 406
BpWRKY23 607
BpWRKY36 468
BpWRKY38 492
BpWRKY46 571
BpWRKY51 492
BpWRKY54 357
BpWRKY55 430
BpWRKY61 441
BpWRKY62 461

B.

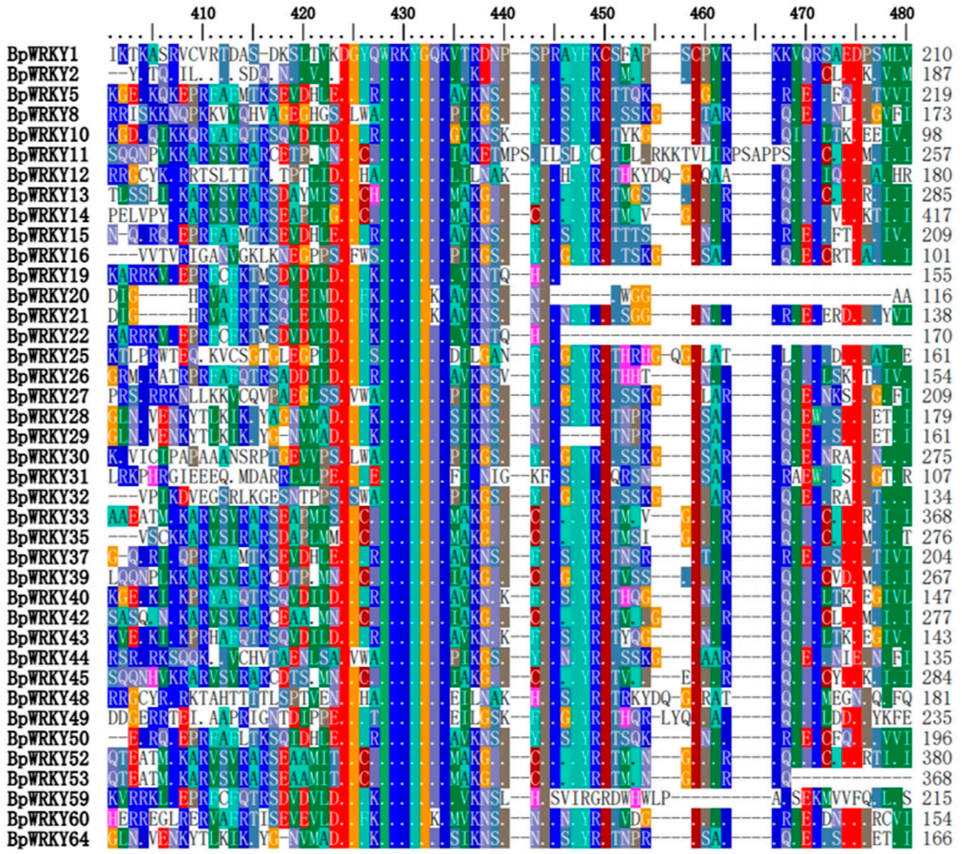

410
420
430
440
450
460
470
480
BpWRKY1 210
BpWRKY2 187
BpWRKY5 219
BpWRKY8 173
BpWRKY10 98
BpWRKY11 257
BpWRKY12 180
BpWRKY13 285
BpWRKY14 417
BpWRKY15 209
BpWRKY16 101
BpWRKY19 155
BpWRKY20 116
BpWRKY21 138
BpWRKY22 170
BpWRKY25 161
BpWRKY26 154
BpWRKY27 209
BpWRKY28 179
BpWRKY29 161
BpWRKY30 275
BpWRKY31 107
BpWRKY32 134
BpWRKY33 368
BpWRKY35 276
BpWRKY37 204
BpWRKY39 267
BpWRKY40 147
BpWRKY42 277
BpWRKY43 143
BpWRKY44 135
BpWRKY45 284
BpWRKY48 181
BpWRKY49 235
BpWRKY50 196
BpWRKY52 380
BpWRKY53 368
BpWRKY59 215
BpWRKY60 154
BpWRKY64 166

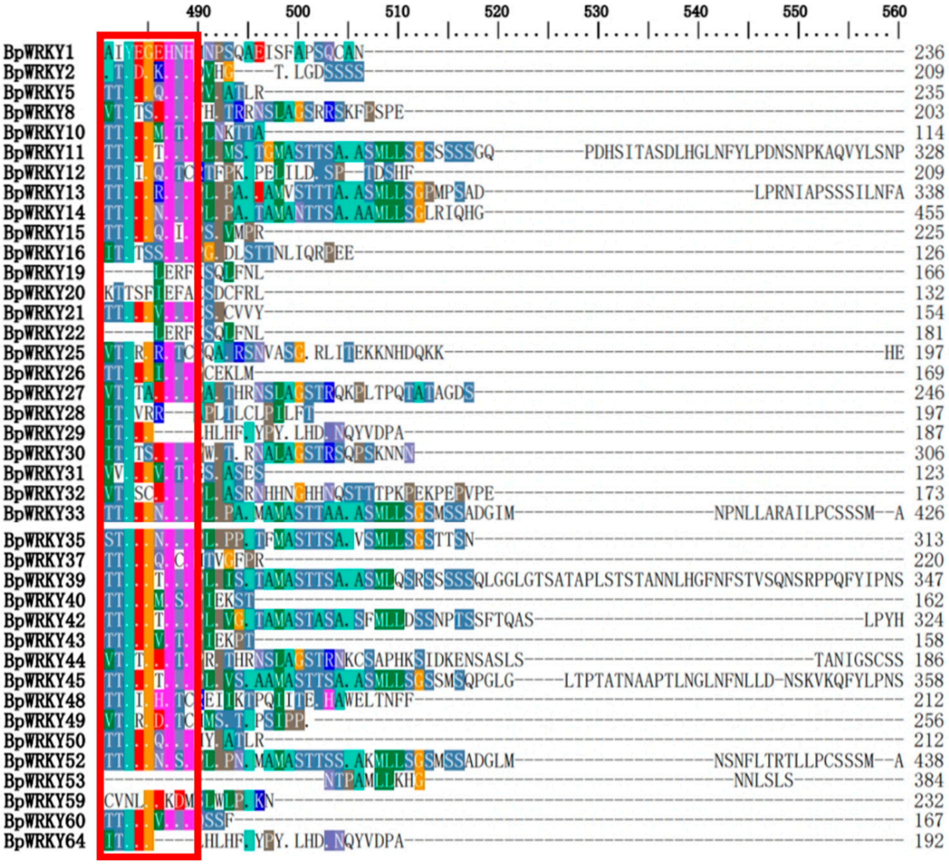
490
500
510
520
530
540
550
560
BpWRKY1
BpWRKY2
BpWRKY5
BpWRKY8
BpWRKY10
BpWRKY11
BpWRKY12
BpWRKY13
BpWRKY14
BpWRKY15
BpWRKY16
BpWRKY19
BpWRKY20
BpWRKY21
BpWRKY22
BpWRKY25
BpWRKY26
BpWRKY27
BpWRKY28
BpWRKY29
BpWRKY30
BpWRKY31
BpWRKY32
BpWRKY33
BpWRKY35
BpWRKY37
BpWRKY39
BpWRKY40
BpWRKY42
BpWRKY43
BpWRKY44
BpWRKY45
BpWRKY48
BpWRKY49
BpWRKY50
BpWRKY52
BpWRKY53
BpWRKY59
BpWRKY60
BpWRKY64

C.

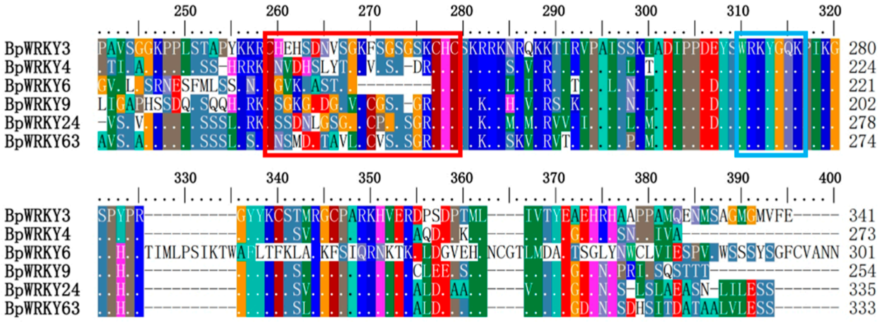
250
260
270
280
290
300
310
320
BpWRKY3
BpWRKY4
BpWRKY6
BpWRKY9
BpWRKY24
BpWRKY63
330
340
350
360
370
380
390
400
BpWRKY3
BpWRKY4
BpWRKY6
BpWRKY9
BpWRKY24
BpWRKY63

D.

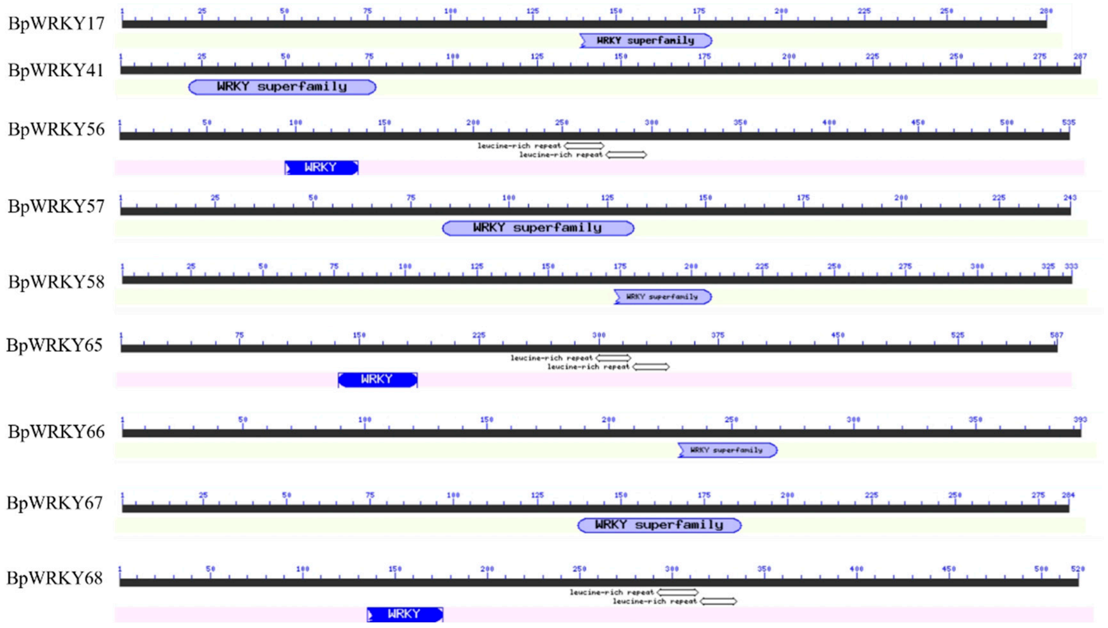

Figure S2. Schematic diagram of the domains of BpWRKYs protein. A: Class I; B: Class II; C: Class III; D: Class IV. The red box indicates zinc-finger domain, and the blue box indicates WRKY domain.
