# Supplementary figures and images for: Genome-Wide Identification and Characterization of WRKY Transcription Factors in Betula platyphylla Suk. and Their Responses to Abiotic Stresses
Source: Int J Mol Sci. 2023 Oct 8;24(19):15000. doi: 10.3390/ijms241915000 (PMC10573109; doi:10.3390/ijms241915000)

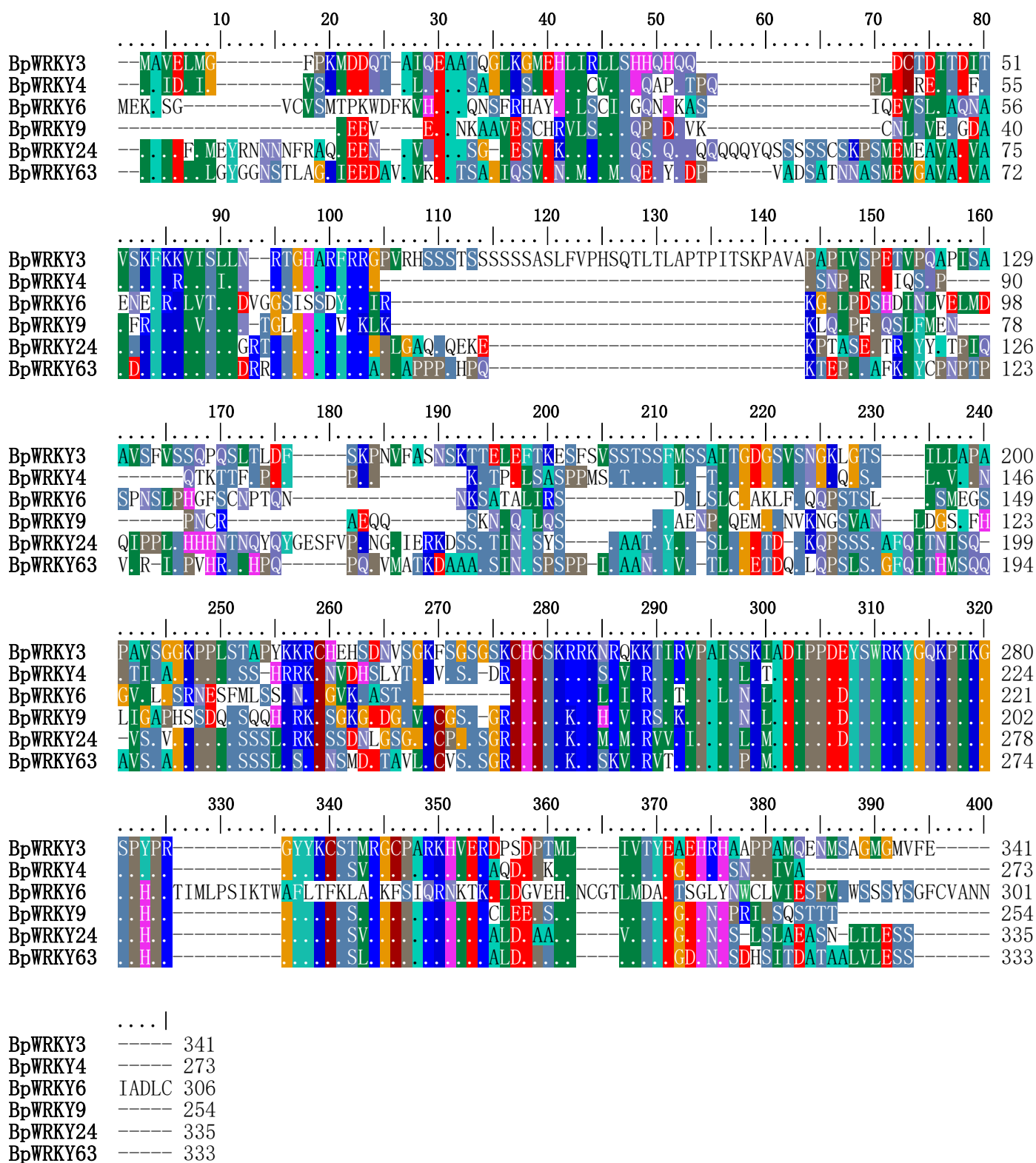

Supplement: Supplementary file 1 [file ijms-24-15000-s001.zip › Figure S1c Multiple sequence alignment analysis of Class ó≤.pdf]

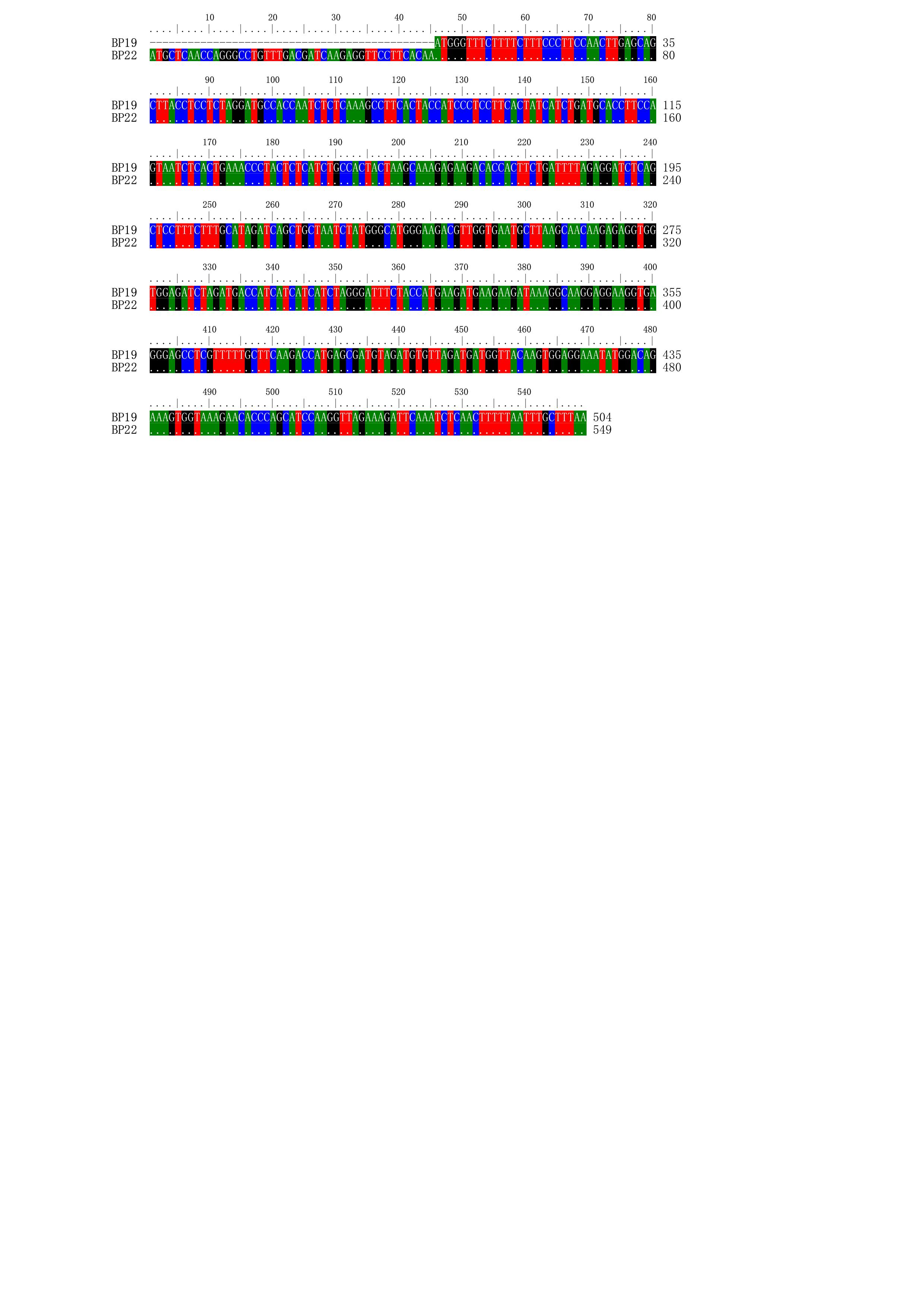

Supplement: Supplementary file 1 [file ijms-24-15000-s001.zip › Figure S3 Multiple sequence alignment analysis of BpWRKY19 and BpWRKY22.jpg]
